# Host defense against oral microbiota by bone-damaging T cells

**DOI:** 10.1038/s41467-018-03147-6

**Published:** 2018-02-16

**Authors:** Masayuki Tsukasaki, Noriko Komatsu, Kazuki Nagashima, Takeshi Nitta, Warunee Pluemsakunthai, Chisa Shukunami, Yoichiro Iwakura, Tomoki Nakashima, Kazuo Okamoto, Hiroshi Takayanagi

**Affiliations:** 1 Department of Immunology, Graduate School of Medicine and Faculty of Medicine, The University of Tokyo, 7-3-1, Hongo, Bunkyo-ku, 113-0033 Tokyo Japan; 2 Department of Molecular Biology and Biochemistry, Biomedical Sciences Major, Graduate School of Biomedical and Health Sciences, Hiroshima University, 1-2-3, Kasumi, Minami-ku, Hiroshima 734-8553 Japan; 3 Research Institute for Biomedical Sciences, Tokyo University of Science, Yamazaki 2669, Noda, Chiba 278-0022 Japan; 4 Department of Cell Signaling, Graduate School of Medical and Dental Sciences, Tokyo Medical and Dental University, Yushima 1-5-45, Bunkyo-ku, Tokyo 113-8549 Japan; 5 Japan Science and Technology Agency (JST), Precursory Research for Embryonic Science and Technology (PRESTO), Yushima 1-5-45, Bunkyo-ku, Tokyo 113-8549 Japan; 6 Japan Agency for Medical Research and Development, Core Research for Evolutional Science and Technology (AMED-CREST), Yushima 1-5-45, Bunkyo-ku, Tokyo 113-8549 Japan; 7 Department of Osteoimmunology, Graduate School of Medicine and Faculty of Medicine, The University of Tokyo, 7-3-1, Hongo, Bunkyo-ku, 113-0033 Tokyo Japan

## Abstract

The immune system evolved to efficiently eradicate invading bacteria and terminate inflammation through balancing inflammatory and regulatory T-cell responses. In autoimmune arthritis, pathogenic T_H_17 cells induce bone destruction and autoimmune inflammation. However, whether a beneficial function of T-cell-induced bone damage exists is unclear. Here, we show that bone-damaging T cells have a critical function in the eradication of bacteria in a mouse model of periodontitis, which is the most common infectious disease. Bacterial invasion leads to the generation of specialized T_H_17 cells that protect against bacteria by evoking mucosal immune responses as well as inducing bone damage, the latter of which also inhibits infection by removing the tooth. Thus, bone-damaging T cells, which may have developed to stop local infection by inducing tooth loss, function as a double-edged sword by protecting against pathogens while also inducing skeletal tissue degradation.

## Abstract (web summary)

IL-17-producing T cells are protective against infection, but the authors of this article previously showed that these cells also contribute to inflammatory bone destruction. Here they show in the context of periodontitis that microbiota-driven Th17-mediated bone destruction may actually be a physiological rather than a pathological process, as associated tooth loss prevents dissemination of oral bacteria.

## Introduction

The interaction between host and microbial communities contributes to human health and disease^1^. The human body surface is mostly covered by an epithelial layer, a physical barrier that functions as the first line of defense against pathogen invasion as well as in response to commensal microbiota^1^. The oral mucosa, however, is exceptional in that the teeth are effectively a trans-mucosal organ, and the interface between each tooth and the mucosa lacks integrity of tight junctions, making it susceptible to infection by oral bacteria^2^. Periodontitis affects >47% adults in the U.S.^3^, and is considered one of the most frequent infectious diseases. Thus, unlike microbiota in other mucosal sites, such as gut and skin, the oral microbiota may have direct and distinct effects on the immune system as well as the health and well-being of the host.

The causal role of the oral microbiome in systemic diseases was first reported in 1891 by the American dentist Willoughby D. Miller^4^. This concept was termed “oral sepsis” and led to the development of a focal infection theory, which was widely accepted until the middle of the twentieth century^5^. However, the theory was discredited and forgotten due to a lack of concrete evidence and ill-advised aggressive tooth extraction^5^. Recent studies have revisited the importance of the oral microbiota based on the close relationship between periodontitis and systemic pathological conditions, including cardiovascular disease, rheumatoid arthritis, adverse pregnancy outcomes, and diabetes^6^. Oral bacteria have been suggested to enter into the systemic circulation via inflamed gingiva and directly affect other organs^6–9^; therefore, the host may have a specialized defense system to protect against oral microbiota, but this mechanism has never been identified.

IL-17 and IL-17-producing T_H_17 cells play an important role in the host defense by inducing anti-bacterial peptides, recruiting neutrophils and promoting local inflammation through cytokines and chemokines^10,11^. T_H_17 cells also contribute to the pathogenesis of various autoimmune diseases by causing prolonged inflammation and tissue damage^10–13^. In autoimmune arthritis, T_H_17 cells function as the exclusive bone-damaging T-cell subset that promotes osteoclastogenesis via the induction of receptor activator of NF-κB ligand (RANKL; encoded by the *Tnfsf11* gene) on synovial fibroblasts through IL-17 production^12,13^. Pathogenic T_H_17 cells in arthritis have been shown to be converted from Foxp3^+^ T cells^14^. The Foxp3^+^ T-cells-derived T_H_17 cells (exFoxp3T_H_17 cells) have a strong pro-inflammatory and pro-osteoclastogenic capacity, contributing to the pathogenesis of autoimmune arthritis^14^. This finding highlighted a crucial role of the plasticity of the CD4^+^ T-cell subsets under various inflammatory disorders^14–19^.

Here, we explore a beneficial function of T-cell-induced bone damage in a periodontitis model, in which exFoxp3T_H_17 cells contribute to protection against bacterial infection as well as induction of bone destruction. We show that periodontitis causes systemic bacterial dissemination in this model, an effect that is ameliorated by tooth extraction. This finding suggests that bone-damaging exFoxp3T_H_17 cells function to stop local infection by removing teeth. Thus, T-cell-mediated bone damage, which has been regarded merely as an adverse secondary effect of inflammation, may be a host defense mechanism against oral bacterial infection.

## Results

### Tooth loss stops systemic dissemination of oral bacteria

**Fig. 1:**
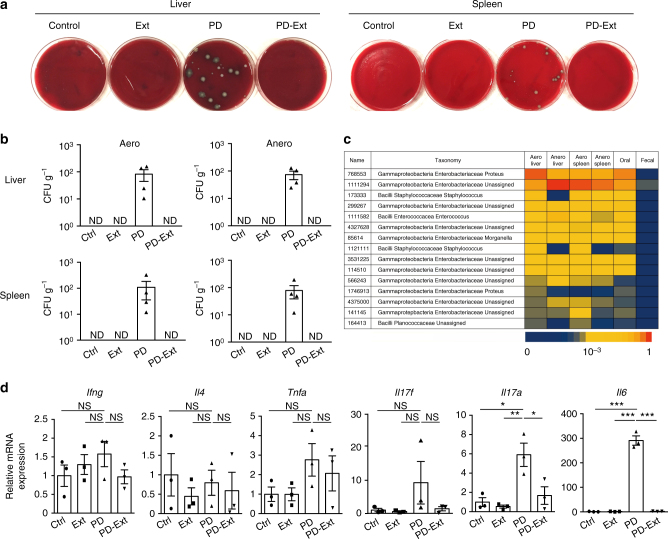
Suppression of oral microbial invasion and local inflammation by tooth extraction. **a** Bacterial colony formation in the culture of the liver and spleen cells from mice subjected to an experimental periodontitis model (PD). This formation was abrogated by tooth extraction (PD-Ext). Colony formation was not observed in the control (Ctrl) or tooth-extracted (Ext) group. Representative pictures of more than three independent experiments are shown. **b** Colony-forming units (CFUs) in aerobic (Aero) and anaerobic (Anero) cultures of liver and spleen cells from mice in Ctrl (*n* = 5), Ext (*n* = 4), PD (*n* = 4), or PD-Ext (*n* = 4) groups, pooled from two independent experiments. **c** 16S sequence analysis of tissue cultures, ligature (Oral), or fecal samples (Fecal) collected from a mouse in the PD group. The top fifteen frequently detected bacterial species in the aerobic liver culture are listed on the left and the sequence frequency (represented by the bottom bar) in each sample is shown. Representative data of more than three independent experiments is presented. **d** Quantitative RT-PCR analysis of inflammatory cytokines in the periodontal tissues collected from mice in the Ctrl, Ext, PD, or PD-Ext groups (*n* = 3). The data were obtained from duplicated experiments. All samples were collected at day 42. All data are shown as the mean ± s.e.m. Statistical analyses were performed using ANOVA with Tukey’s multiple-comparison test. *P < 0.05; **P < 0.01; ***P < 0.005; ND, not detected; NS, not significant

Periodontitis patients often develop bacteremia^7,8^, however, there has been little experimental evidence reported that shows oral bacteria translocate to other organs using animal models. We used a mouse model of periodontitis^20^ in which the placement of silk ligature around tooth leads to an accumulation of oral bacteria followed by inflammation and bone destruction. Livers, spleens, and the periodontal tissues were collected and analyzed after 42 days of periodontitis induction (Supplementary Fig. 1a). Notably, we detected bacterial colony formation in a culture of liver and spleen cells after persistent ligature placement around the tooth (Fig. 1a, b). The bacterial species detected in the liver and spleen were found in the oral cavity as well, but not in fecal samples (Fig. 1c), indicating systemic dissemination of oral bacteria during the breakdown of the oral barrier. Two weeks after periodontitis induction, the infected tooth became loose and easily dissociated from the jawbone due to osteoclastic resorption of the tooth-supporting bone (Supplementary Fig. 1b, c). We extracted the infected tooth and analyzed the local and systemic effects in an effort to elucidate the effects of tooth loss on the host. After tooth loss, the socket healed and became covered by the oral mucosa in less than 4 weeks (Supplementary Fig. 1), ending the systemic dissemination of oral bacteria (Fig. 1a, b) as well as the local inflammation in the oral mucosa (Fig. 1d). These results suggest that removal of an infected tooth by enhanced osteoclastic bone resorption is a host defense mechanism against the invasion of oral microbiota.

### T_H_17 cell accumulation induced by oral microbiota

**Fig. 2:**
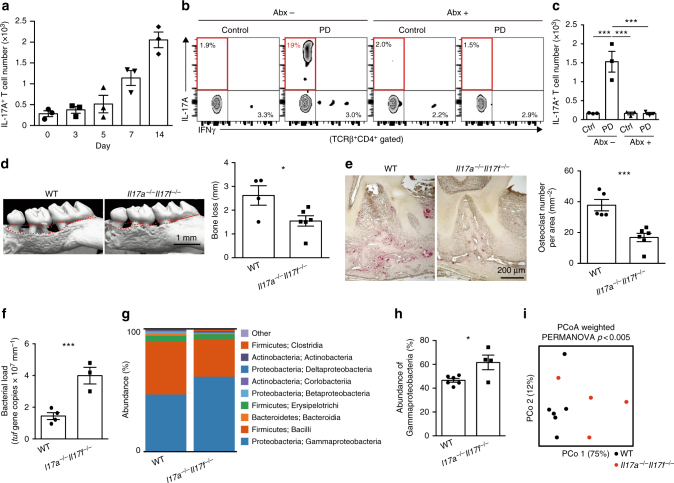
T_H_17 cells protect against the invasion of oral microbiota and induce bone damage. **a** Number of IL-17A^+^CD4^+^TCRβ^+^ cells in the periodontal tissues at various time points after the ligature placement (*n* = 3). Representative data of two independent experiments is presented. **b**, **c** Effects of an antibiotic cocktail (Abx; ampicillin 1 mg ml^−1^, streptomycin 5 mg ml^−1^, and colistin 1 mg ml^−1^ in drinking water) on the accumulation of T_H_17 cells. Mice were treated with Abx from 1 week before the ligature placement. The frequency (**b**) and number (**c**) of IL-17A^+^CD4^+^TCRβ^+^ cells in the periodontal tissues were analyzed 7 days after the ligature placement (*n* = 3). PD: periodontitis. **d** Micro-CT analysis of periodontitis-induced bone loss in the wild-type (*n* = 4) or *Il17a*^−/−^*Il17f*^−/−^ mice (*n* = 6), pooled from three independent experiments. The upper red dotted line indicates the cementoenamel junction and the lower red dotted line indicates the alveolar bone crest in the left panel. **e** Osteoclast number in the maxilla of the wild-type (*n* = 5) or *Il17a*^−/−^*Il17f*^−/−^ mice (*n* = 6), pooled from two independent experiments. **f** Total bacterial load in ligatures collected from wild-type (*n* = 4) or *Il17a*^−/−^*Il17f*^−/−^ mice (*n* = 3). Representative data of two independent experiments is presented. **g** Oral bacterial composition (major phylum; class) of the wild-type (*n* = 6) or *Il17a*^−/−^*Il17f*^–/–^mice (*n* = 4). **h** Abundance of γ-proteobacteria in the oral bacteria of the wild-type (*n* = 6) or *Il17a*^−/−^*Il17f*^−/−^mice (*n* = 4). **i** Differences in the bacterial composition between the wild-type (*n* = 6) and *Il17a*^−/−^*Il17f*^−/−^mice (*n* = 4). Principal coordinate analysis (PCoA) and permutational ANOVA (PERMANOVA) comparisons of the weighted UniFrac distances are shown. PCo1: principal coordinate 1; PCo2: principal coordinate 2. The data were pooled from two independent experiments (**g**–**i**). All data are shown as the mean ± s.e.m. Statistical analyses were performed using ANOVA with Tukey’s multiple-comparison test (**c**), Student’s *t*-test (**d**–**f** and **h**) or PERMANOVA of the weighted UniFrac distances (**i**). *P < 0.05; ***P < 0.005

We thus hypothesized that the immune cells that direct osteoclastic bone erosion have a key role in the host defense against oral infection. Since CD4^+^ T cells have been shown to be essential for the periodontitis-induced bone loss[21], we investigated which subsets of CD4^+^ T cells were increased during oral infection. Among the CD4^+^ T-cell subsets, T_H_17 cells exclusively accumulated in the oral mucosa and draining lymph nodes during oral infection (Fig. 2a–c, Supplementary Fig. 2), consistent with high expression of T_H_17-related cytokines *Il17a* and *Il6* in the oral mucosa of periodontitis-induced mice (Fig. 1d). In contrast, the frequency of Foxp3^+^ T cells was significantly decreased in the draining lymph nodes during periodontitis (Supplementary Fig. 2c). The number of neutrophils was significantly increased in the periodontal lesion, whereas the number of other immune cell populations (macrophages, dendritic cells, B cells or γδT cells) was not increased, suggesting that the T_H_17 response is dominant in the inflamed gingiva (Supplementary Fig. 3). It has been reported that the physiological maintenance of T_H_17 cells in the oral tissue is not dependent on the microbiota but mastication-induced mechanical damage^22^. However, treatment with a broad-spectrum antibiotic cocktail completely inhibited the accumulation of T_H_17 cells in the oral mucosa and draining lymph nodes in the periodontitis model (Fig. 2b, c and Supplementary Fig. 2e–g), indicating that the T_H_17 cell accumulation in the periodontal lesion is heavily dependent on the oral microbiota.

### Induction of periodontal bone destruction by T_H_17 cells

T_H_17 cells are the exclusive osteoclastogenic T-cell subset that promotes osteoclastogenesis via the induction of RANKL on mesenchymal cells, such as synovial fibroblasts, through IL-17 production^12,13^. However, the role of T_H_17 cells in oral infection-associated bone damage is obscure^3^. Despite the high expression of IL-17A in inflamed gingiva (Fig. 1d and Supplementary Fig. 4b), periodontitis-induced bone loss in *Il17a*^–/–^ mice was comparable with that in wild-type mice (Supplementary Fig. 4a). The expression of IL-17F was increased in the inflamed gingiva in *Il17a*^–/–^ mice (Supplementary Fig. 4c), potentially compensating for the IL-17A deficiency. Therefore, we used *Il17a*^–/–^*Il17f*^–/–^ mice to investigate the role of T_H_17 cells and found that periodontal bone loss was significantly inhibited in *Il17a*^–/–^*Il17f*^–/–^mice (Fig. 2d, e). IL-17 is also produced by γδT cells^23^; however, periodontitis-induced bone loss was suppressed in *Tcra*^–/–^ mice but not *Tcrd*^–/–^ mice (Supplementary Fig. 4d), suggesting that T_H_17 cells rather than γδT cells are involved in the bone damage during oral infection.

### Regulation of oral microbiota by T_H_17 cells

Since IL-17 plays a key role in host defense against extracellular bacterial infections^10^, we monitored the quantity and composition of oral bacteria that accumulated at the inflammatory site in *Il17a*^–/–^*Il17f*^–/–^ mice. The total amount of bacterial DNA collected from the periodontal lesion was significantly increased in *Il17a*^–/–^*Il17f*^−/–^ mice (Fig. 2f). Moreover, 16 S sequence analysis revealed that the composition of the oral bacteria was significantly altered in *Il17a*^–/–^*Il17f*^–/–^ mice (Fig. 2g–i). The frequency of γ-proteobacteria, which predominantly colonizes peripheral tissues during the dissemination of oral bacteria (Fig. 1c), was increased in the oral cavity of *Il17a*^–/–^*Il17f*^–/–^ mice (Fig. 2g, h). During the course of periodontitis in wild-type mice, the abundance of γ-proteobacteria in the inflammatory site was decreased at day 7, when T_H_17 cells started to accumulate in the periodontal lesion (Supplementary Fig. 5). These results suggest that T_H_17 cells play a key role in host defense against the invasion of oral bacteria via the elimination of oral bacteria in addition to induction of bone loss.

### Foxp3^+^ T cells convert into T_H_17 cells during oral infection

Next, we explored the origin of T_H_17 cells that accumulated in the periodontal lesion. T_H_17 cells are abundantly present in the small intestine lamina propria, especially in the terminal ileum^24–26^. Thus, we asked whether T_H_17 cells migrated from the small intestine to the periodontal tissues during oral infection. We utilized ROSA-CAG-lox-stop-lox-hKikGR × *Vav*-iCre mice in which violet light (436 nm) induces photoconversion of the KikGR protein from green (KikGR-green) to red (KikGR-red) in *Vav*-iCre-expressing haematopoietic lineage cells^27^. To track T_H_17 cell migration from the small intestine to the periodontal tissue, the mucosa of the terminal ileum was exposed to violet light (436 nm) under anesthesia at day 5 after periodontitis induction (Supplementary Fig. 6a). The mesenteric lymph nodes, cervical lymph nodes, and periodontal tissues were collected and analyzed at day 7, when T_H_17 cells markedly accumulated in the periodontal tissues (Fig. 2a and Supplementary Fig. 6a). KikGR-red^+^ T cells were detected in the mesenteric lymph nodes, indicating that T cells migrated from the terminal ileum mucosa to the mesenteric lymph nodes (Supplementary Fig. 6b). In contrast, T_H_17 cells in the periodontal tissues or the draining lymph nodes were KikGR-red negative, indicating that T_H_17 cells in the inflamed gingiva were not originated from the small intestine (Supplementary Fig. 6c). These results suggested that T_H_17 cells are locally expanded and/or differentiated in the periodontal lesion during oral infection.

It has been reported that the plasticity of the CD4^+^ T-cell subsets has a key role under various inflammatory conditions^14–19^. Foxp3^+^ T-cells-derived T_H_17 cells, exFoxp3T_H_17 cells, have been shown to crucially contribute to the pathogenesis of asthma^17^ and rheumatoid arthritis^14^. Interestingly, exFoxp3T_H_17 cells were shown to have the strongest pro-osteoclastogenic capacity among the CD4^+^ T-cell subsets in vitro^14^. However, the role of exFoxp3T_H_17 cells in host immunity is unknown. We next investigated whether exFoxp3T_H_17 cells contribute to the host defense against oral bacteria. To monitor the loss of Foxp3 expression in vivo, we crossed *Foxp3*-GFP-Cre mice with ROSA26-YFP reporter mice, in which exFoxp3T cells are marked as GFP^–^YFP^+^ T cells^14^. We found that exFoxp3T cells in periodontitis-induced mice are characterized by a higher expression of RANKL and CCR6 than GFP^–^YFP^–^ T cells (conventional T cells) (Supplementary Fig. 7a). exFoxp3T cells in periodontitis-induced mice expressed regulatory T (T_reg_) cell signature molecules such as GITR, KLRG1, FR4, and Nrp1 to a similar extent to GFP^+^YFP^+^ T cells while GFP^–^YFP^–^ T cells expressed these genes at a lower level (Supplementary Fig. 7c). exFoxp3T cells also expressed OX40, CD25, CD39, CD103, Helios, and CTLA-4, although to a lesser extent compared to GFP^+^YFP^+^ T cells (Supplementary Fig. 7b, c). These characteristics of exFoxp3T cells in periodontitis-induced mice are similar to those previously reported in arthritic mice^14^.

**Fig. 3:**
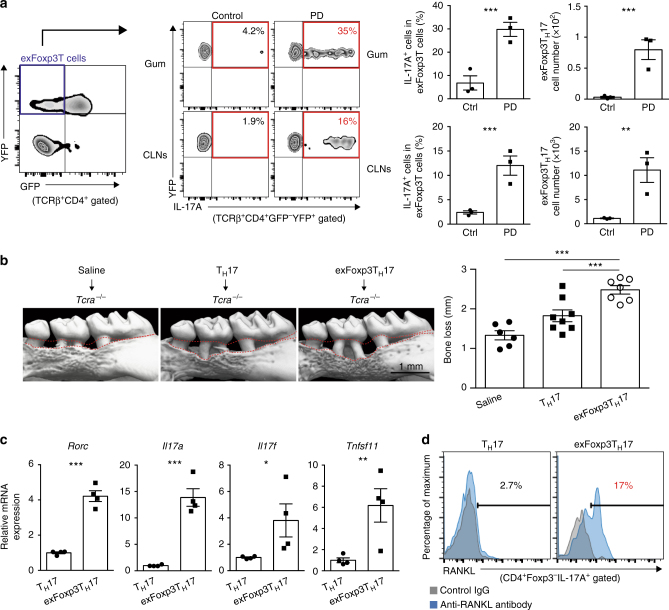
A crucial role for exFoxp3T_H_17 cells in the bone destruction during oral infection. **a** Frequency and number of exFoxp3T_H_17 cells in the periodontal tissues (Gum) and the cervical lymph nodes (CLNs) in control or periodontitis-induced mice 7 days after the ligature placement (*n* = 3). Representative data of more than three independent experiments is presented. PD: periodontitis. **b** Effects of adoptive transfer of T_H_17 cells (*n* = 8) or exFoxp3T_H_17 cells (*n* = 7) on periodontitis-induced bone loss compared to the saline group (*n* = 6). The data were pooled from more than three independent experiments. The upper red dotted line indicates the cementoenamel junction and the lower red dotted line indicates the alveolar bone crest in the left panel. All data are shown as the mean ± s.e.m. **c** Quantitative RT-PCR analysis of *Rorc*, *Il17a*, *Il17f*, and *Tnfsf11* transcripts in T_H_17 cells or exFoxp3T_H_17 cells (*n* = 4). The data were pooled from two independent experiments. **d** FACS profiles of RANKL expression in T_H_17 cells or exFoxp3T_H_17 cells. Representative data of more than three independent experiments is shown. Statistical analyses were performed using Student’s *t*-test (**a**, **c**), ANOVA with Tukey’s multiple-comparison test (**b**). *P < 0.05; **P < 0.01; ***P < 0.005

Fate mapping analysis further showed that the frequency and number of exFoxp3T_H_17 cells to be markedly increased in the oral mucosa and draining lymph nodes during oral infection (Fig. 3a). Conversely, the frequency or number of IFNγ-expressing exFoxp3T cells (exFoxp3T_H_1 cells) or IL-4-expressing exFoxp3T cells (exFoxp3T_H_2 cells) was not increased (Supplementary Fig. 8). Adoptive transfer of exFoxp3T_H_17 cells into *Tcra*^–/–^ mice significantly promoted periodontitis-induced bone loss compared with the transfer of conventional T_H_17 cells or saline controls (Fig. 3b and Supplementary Fig. 9). Interestingly, exFoxp3T_H_17 cells in periodontitis-induced mice had much higher expression levels of effecter molecules such as *Rorc*, *Il17a*, *Il17f*, and *Tnfsf11* than conventional T_H_17 cells (Fig. 3c). exFoxp3T_H_17 cells expressed high amounts of membrane-bound RANKL even in the absence of in vitro stimulation (Fig. 3d).

### Identification of the source of RANKL in periodontitis

**Fig. 4:**
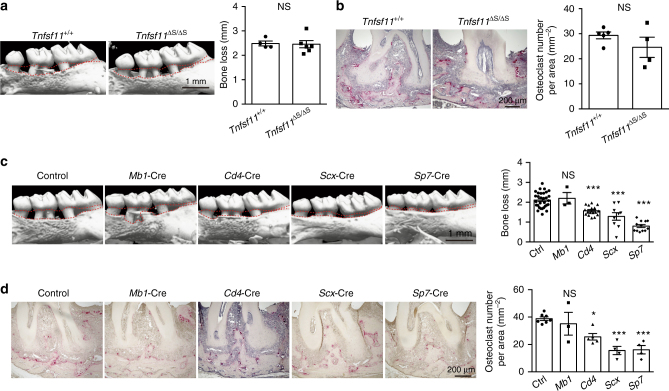
Osteoblasts and periodontal ligament cells are the major source of RANKL in periodontitis. **a** Micro-CT analysis of periodontitis-induced bone loss in *Tnfsf11*^*+/+*^ mice (*n* = 4) or *Tnfsf11*^ΔS/ΔS^ mice (*n* = 6). The upper red dotted line indicates the cementoenamel junction and the lower red dotted line indicates the alveolar bone crest. **b** Histological analysis of periodontitis-induced osteoclast development in *Tnfsf11*^*+/+*^ mice (*n* = 5) or *Tnfsf11*^ΔS/ΔS^ mice (*n* = 4). **c** Micro-CT analysis of periodontitis-induced bone loss in mice in which RANKL was specifically deleted in B cells (*Mb1*-Cre) (*n* = 3), T cells (*Cd4*-Cre) (*n* = 17), periodontal ligament cells (*Scx*-Cre) (*n* = 9) or osteoblastic cells (*Sp7*-Cre) (*n* = 12) compared to control mice (*n* = 31). The upper red dotted line indicates the cementoenamel junction and the lower red dotted line indicates the alveolar bone crest in the left panel. **d** Osteoclast number in the maxilla of mice in which RANKL was specifically deleted in B cells (*Mb1*-Cre) (*n* = 3), T cells (*Cd4*-Cre) (*n* = 5), periodontal ligament cells (*Scx*-Cre) (*n* = 4) or osteoblastic cells (*Sp7*-Cre) (*n* = 4) compared to control mice (*n* = 7) evaluated by TRAP staining. The data were pooled from more than three independent experiments (**c**, **d**). All data are shown as the mean ± s.e.m. Statistical analyses were performed using Student’s *t*-test (**a**,** b**), ANOVA with Dunnett’s multiple-comparison test (**c**, **d**). *P < 0.05; ***P < 0.005; NS, not significant

Although RANKL is mainly functional in its membrane-bound form^28^, the soluble RANKL produced by activated B cells and T cells has been proposed to play a major role in periodontal bone loss^29^. Which type of RANKL is critical and which cell type(s) is the main source of RANKL in periodontitis-induced bone damage? To reveal the role of soluble RANKL to periodontal bone loss, we exploited *Tnfsf11*^ΔS/ΔS^ mice, in which the soluble form of RANKL is absent and thus only membrane-bound RANKL exists^30^. We found that there was no difference in periodontitis-induced bone loss between *Tnfsf11*^ΔS/ΔS^ mice and wild-type mice (Fig. 4a, b), showing that the contribution of soluble RANKL is negligible. Thus, RANKL-producing cells should directly interact with osteoclast precursors in a cell–cell contact manner. In situ hybridization revealed that RANKL was expressed in mesenchymal cells including osteoblastic cells and periodontal ligament cells as well as hematopoietic cells adjacent to alveolar bone during periodontal inflammation (Supplementary Fig. 10).

We crossed *Tnfsf11*^*flox/flox*^ mice with various Cre lines specific to B cells, T cells, osteoblastic cells and periodontal ligament cells to investigate the source of RANKL in periodontitis^31^. Since RANKL on osteoblastic cells is essential for tooth eruption^32^, we used a *Sp7*-tTA-tetO-Cre (*Sp7*-Cre) system in which Cre recombinase is expressed only when a tetracycline-controlled transactivator (tTA) binds to a tetracycline responsive element (tetO) in the absence of doxycycline (Dox)^33^. *Sp7*-Cre mice crossed with RANKL-floxed mice were treated from the prenatal period with Dox, which was withdrawn at the age of 3 weeks. Periodontitis was induced at the age of 8 weeks and alveolar bone was analyzed 10 days after the ligature placement in all groups. We found that periodontitis-induced bone loss and osteoclast number were markedly suppressed when RANKL was deleted in osteoblastic cells (*Sp7*-Cre) and periodontal ligament cells (*Scx*-Cre) (Fig. 4c, d). Osteoblastic cells and periodontal ligament cells reportedly express RANKL in response to IL-17 (refs. ^34,35^). The periodontitis-induced bone loss and osteoclast number were significantly reduced, albeit to a lesser extent, when RANKL was deleted in T cells (*Cd4*-Cre) but not B cells (*Mb1*-Cre) (Fig. 4c, d). Thus, it is likely that exFoxp3T_H_17 cells contribute to bone loss by inducing RANKL expression mainly on osteoblastic cells and periodontal ligament cells via IL-17 production.

### IL-6 induces the generation of exFoxp3T_H_17 cells

**Fig. 5:**
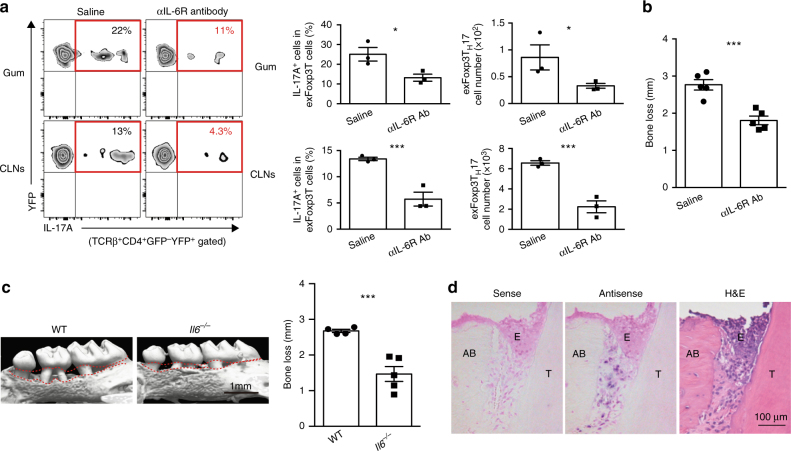
IL-6 facilitates the generation of exFoxp3T_H_17 cells during periodontal infection. **a** Frequency and number of exFoxp3T_H_17 cells in periodontal tissues (Gum) and cervical lymph nodes (CLNs) in periodontitis-induced mice treated with saline or anti-IL-6R antibody (Ab) 7 days after the ligature placement (*n* = 3). Two milligrams of anti-IL-6R Ab (MR16-1) were injected intraperitoneally after 2 days of the ligature placement. Representative data of two independent experiments is shown. **b** Periodontitis-induced bone loss in control mice or anti-IL-6R Ab-treated mice (*n* = 5), pooled from two independent experiments. **c** Micro-CT analysis of periodontitis-induced bone loss in wild-type mice (*n* = 4) or *Il6*^−/−^ mice (*n* = 5). The upper red dotted line indicates the cementoenamel junction and the lower red dotted line indicates the alveolar bone crest. **d** In situ hybridization of *Il6* mRNA in periodontitis-induced wild-type mice 3 days after the ligature placement. Representative data of more than three independent experiments is shown. H&E, haematoxylin and eosin stain; E, epithelium; T, tooth; AB, alveolar bone. All data are shown as the mean ± s.e.m. Statistical analyses were performed using Student’s *t*-test. *P < 0.05; ***P < 0.005

The conversion from Foxp3^+^ T cells into exFoxp3T_H_17 cells was shown to be dependent on the IL-6 signaling pathway in vitro^14^, so we investigated whether the development of exFoxp3T_H_17 cells at the oral barrier is also mediated by IL-6 in vivo. Treatment with anti-IL-6 receptor (IL-6R) antibody significantly inhibited the generation of exFoxp3T_H_17 cells (Fig. 5a) as well as bone loss in periodontitis-induced mice (Fig. 5b). IL-6-deficient mice were also resistant to periodontal bone loss (Fig. 5c). Periodontitis-induced *Il17a* expression in the oral mucosa was completely inhibited in *Il6*^*–/–*^ mice (Supplementary Fig. 11a). The expression of *Rorc* in CD4^+^ T cells sorted from cervical lymph nodes was increased during periodontitis in wild-type mice, but not in *Il6*^*–/–*^ mice (Supplementary Fig. 11b). These results indicated that IL-6 signaling is essential for the induction of T_H_17 cells and exFoxp3T_H_17 cells during oral infection. Although gingival epithelial cells are reportedly a major producer of IL-6 in mastication-induced T_H_17 cell development in the oral mucosa^22^, IL-6 expression was detected at the periodontal ligament site but not the epithelium in the periodontitis model (Fig. 5d).

### Regulation of oral bacterial infection by exFoxp3T_H_17 cells

**Fig. 6:**
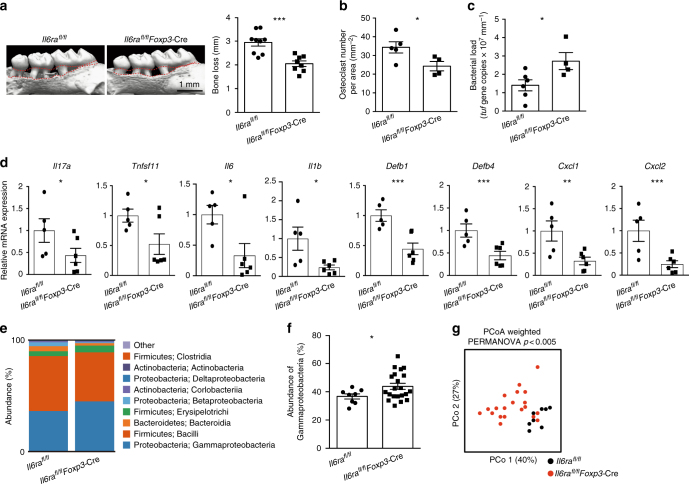
Induction of anti-bacterial response and bone damage by exFoxp3T_H_17 cells. **a** Micro-CT analysis of periodontitis-induced bone loss in *Il6ra*^*flox/flox*^ mice (*n* = 9) or *Il6ra*^*flox/flox*^ *Foxp3*-Cre mice (*n* = 8). The upper red dotted line indicates the cementoenamel junction and the lower red dotted line indicates the alveolar bone crest. **b** Number of osteoclasts in the maxilla of periodontitis-induced *Il6ra*^*flox/flox*^ mice (*n* = 5) or *Il6ra*^*flox/flox*^ *Foxp3*-Cre mice (*n* = 4), pooled from two independent experiments. **c** Total bacterial load determined by analyzing the *tuf* gene copy number in ligatures collected from *Il6ra*^*flox/flox*^ mice (*n* = 6) or *Il6ra*^*flox/flox*^ *Foxp3*-Cre mice (*n* = 4). **d** Quantitative RT-PCR analysis of *Il17a*, *Tnfsf11*, *Il6*, *Il1b*, *Defb1*, *Defb4*, *Cxcl1*, and *Cxcl2* transcripts in the periodontal tissues collected from periodontitis-induced *Il6ra*^*flox/flox*^ mice (*n* = 5) or *Il6ra*^*flox/flox*^ *Foxp3*-Cre mice (*n* = 6). The data were obtained from duplicated experiments. **e** Bacterial composition (major phylum; class) of DNA collected from ligatures of *Il6ra*^*flox/flox*^ mice (*n* = 8) or *Il6ra*^*flox/flox*^ *Foxp3*-Cre mice (*n* = 20). **f** Abundance of γ-proteobacteria in the bacterial DNA collected from the ligatures of *Il6ra*^*flox/flox*^ mice (*n* = 8) or *Il6ra*^*flox/flox*^ *Foxp3*-Cre mice (*n* = 20). **g** Differences in the bacterial communities between *Il6ra*^*flox/flox*^ mice (*n* = 8) and *Il6ra*^*flox/flox*^ *Foxp3*-cre mice (*n* = 20). PCo1: principal coordinate 1; PCo2: principal coordinate 2. The data were pooled from more than three independent experiments (**e**–**g**). PERMANOVA comparisons of the weighted UniFrac distances are shown. All data are shown as the mean ± s.e.m. Statistical analyses were performed using Student’s *t*-test (**a**–**d** and **f**) or PERMANOVA of the weighted UniFrac distances (**g**). *P < 0.05; **P < 0.01; ***P < 0.005

Finally, we crossed *Il6ra*^*flox/flox*^ mice with *Foxp3*-Cre mice in order to specifically deplete exFoxp3T_H_17 cells^17^. Similar to the phenotype seen in *Il17a*^–/–^*Il17f*^–/–^ mice (Fig. 2d–i), *Il6ra*^*flox/flox*^ *Foxp3*-Cre mice displayed milder bone loss but increased bacterial load compared with control mice during periodontal infection (Fig. 6a–c). In addition to *Il17a* and *Tnfsf11*, the expression levels of inflammatory cytokines *Il6* and *Il1b*, the antimicrobial peptides *Defb1* and *Defb4*, and the neutrophil chemo-attractants *Cxcl1* and *Cxcl2* were significantly suppressed in the absence of exFoxp3T_H_17 cells during periodontal infection (Fig. 6d). Furthermore, the bacterial composition was significantly altered in the oral cavity of *Il6ra*^*flox/flox*^ *Foxp3*-Cre mice (Fig. 6e–g). The frequency of γ-proteobacteria was increased in the *Il6ra*^*flox/flox*^ *Foxp3*-Cre mice (Fig. 6e, f), consistent with our observations in the *Il17a*^–/–^*Il17f*^–/–^ mice (Fig. 2g, h). Since IL-17 has been shown to induce the expression of defensins and chemokines in intestinal epithelial cells to regulate gut microbiota^10^, it is likely that exFoxp3T_H_17 cells control the quantity and quality of oral bacteria via the induction of antimicrobial peptides and chemokines in the gingival epithelial cells by IL-17 during oral infection. Collectively, the data show a crucial role for the conversion of Foxp3^+^ T cells into exFoxp3T_H_17 cells in host immunity against oral microbiota (Supplementary Fig. 12).

## Discussion

Here, we show that bone-damaging T cells, T_H_17 cells converted from Foxp3^+^ T cells, orchestrate the host defense against oral microbiota by regulating both osteoclastic bone resorption and antimicrobial immunity. Needless to say, the teeth comprise an essential, non-substitutable organ that are not typically extracted to prevent infection in modern times since oral infection is controllable by dental interventions^36^. In periodontitis patients, teeth with severe infections are eventually lost due to the resorption of tooth-supporting bone, resulting in a complete resolution of infection and inflammation. In contrast, osteopetrotic patients with periodontal infection develop severe osteomyelitis of the jaw, which is treated by tooth extraction and antibiotic treatment^37^. These observations support the concept that inflammatory bone loss is crucial for the termination of oral infection.

One of the most serious side effects of anti-bone resorptive drugs (e.g., bisphosphonate and anti-RANKL antibody) is osteonecrosis of the jaw (ONJ)^38^. Intriguingly, the osteonecrosis is observed only in the jaw, probably due to the unique feature of the jawbone where teeth are embedded and easily exposed to the oral bacterial invasion. Tooth extraction has been thought as a potential trigger for the onset of ONJ^38^. However, recent studies have shown that local infection, but not tooth extraction itself, is a key risk factor for the development of ONJ^39,40^. It has recently been suggested that tooth extraction aiming at the eradication of a local infection may decrease the risk for the development of ONJ[39]. The concept of tooth loss as the host defense mechanism presented in this study may provide additional insights into the understanding of the pathogenesis of ONJ.

T_H_17 cells are abundantly present in the human periodontal lesion^3^ and shown to be the major cellular source of IL-17 in the periodontal tissues of periodontitis patients^41^. The levels of IL-17 in the periodontal tissues correlate with the severity of periodontitis in humans^42^. Importantly, Foxp3^+^IL-17^+^ cells, which apparently are in the transition state during the conversion, were observed in the periodontal tissues obtained from severe periodontitis patients^43^, suggesting that exFoxp3T_H_17 cells may play a key role in the pathogenesis of human periodontitis.

The generation of exFoxp3T_H_17 cells in the oral mucosa is heavily dependent on IL-6 signaling pathway (Fig. 5a). A clinical study showed that tocilizumab, an IL-6R inhibitor, significantly ameliorated periodontal inflammation^44^. The IL-6 messenger RNA (mRNA) was highly expressed in the periodontal ligament cells during periodontitis (Fig. 5d). It has been reported that IL-6 production by periodontal ligament fibroblasts is stimulated by bacteria (e.g., *Porphyromonas gingivalis*, *Prevotella intermdedia*, *Fusobacterium nucleatum*, *Aggregatibacter actinomycetemcomitans* and *Escherichia coli*) and pathogen-associated molecular patterns (PAMPs) (e.g., lipopolysaccharides (LPS), peptidoglycan (PGN), muramyl dipeptide (MDP), l-Ala-γ-d-Glu-mDAP (Tri-DAP) and Pam3CysSerLys4 (Pam3CSK4))^45–50^. Thus, oral bacteria and their components may directly stimulate IL-6 production by periodontal ligament fibroblasts during periodontitis. Collectively, fine-tuning of bone-damaging T cells with an appropriate control of oral infection may be a promising strategy to prevent periodontitis-induced bone and tooth loss.

The mechanisms of inflammatory bone destruction have been intensively investigated in the field of rheumatoid arthritis^12^. In autoimmune arthritis, pathogenic T_H_17 cells that induce bone destruction are also causative of autoimmune inflammation. However, it has never been properly elucidated why the activation of host immunity is designed so as to induce bone damage. Among the inflammatory bone disorders, alveolar bone loss due to oral infection is most commonly observed in the vertebrates, including ancient reptiles^51^, in which teeth are constantly replaced. Although teeth are essential for eating and fighting in mammals, we speculate that inflammatory bone erosion followed by tooth loss is a primitive host defense mechanism to prevent prolonged bacterial invasion, a mechanism, which has been evolutionarily conserved from animals with replaceable teeth to humans. Indeed, the *Tnfsf11* and *Il17a* genes are widely conserved across species ranging from jawed fishes to humans^52^.

The symbiotic relationship between gut microbiota and host is tightly regulated by the intestinal barrier function^1^. In contrast, oral microbiota easily flow into the bloodstream via the inflamed gingiva. Thus, the oral microbiota have the capacity to impact systemic health in a manner distinctly different from the gut microbiota. Our study highlights a unique host defense system against microorganisms in which the immune and skeletal systems cooperate, extending the horizon of host–microbiome interactions.

## Methods

### Mice

All animals were maintained under specific pathogen-free conditions, and all experiments were performed with the approval of the Institutional Review Board at The University of Tokyo. C57BL/6 mice were purchased from CLEA Japan. *Foxp3*-YFP-Cre mice, *Il6ra* ^*flox/flox*^ mice were obtained from the Jackson Laboratory. *Foxp3*^*hCD2*^ knock-in mice^14,53^, *Il17a*-GFP knock-in mice^14^, *Foxp3*-GFP-Cre mice^14^, ROSA26-loxP-Stop-loxP-YFP reporter mice^14^, *Il17a*^–/–^ mice^10^, *Il17f*^–/–^ mice^10^, *Tnfsf11* ^*flox/flox*^ mice^30^, *Tnfsf11*^ΔS/ΔS^ mice^30^, *Scx*-Cre mice^54,55^, *Cd4*-Cre mice^56^, *Mb1*-Cre mice^57^, *Sp7-*Cre mice^33^, *Il6*^–/–^ mice^33^, *Tcrd*^–/–^ mice^23^, *Tcra*^–/–^ mice^58^, ROSA-CAG-lox-stop-lox-hKikGR mice^27^, and *Vav*-iCre mice^59^ were described previously. Eight- to 16-week-old sex-matched mice were used for all of the experiments unless otherwise noted.

### Ligature-induced periodontitis model

To evaluate the periodontitis-induced bone loss, a 5-0 silk ligature was tied around the maxillary left second molar and the contralateral tooth was left unligated to serve as the baseline control, as described previously^20^. The distance between the cementoenamel junction and alveolar bone crest (CEJ–ABC distance) was measured for six predetermined maxillary sites on both the buccal and palatal sides^20^. The mice were sacrificed and analyzed 10 days after placement of the ligature unless otherwise indicated. The maxillae were subjected to micro-CT and histological analyses, respectively. For micro-CT analysis, the maxillae were fixed with 70% ethanol until analysis. Micro-CT scanning was performed with a ScanXmate-A100S Scanner (Comscantechno). Three-dimensional microstructural image data were reconstructed and structural indices were calculated using TRI/3D-BON software (RATOC). For histological analyses, the maxillae fixed by 4% paraformaldehyde underwent decalcification in OSTEOSOFT ^®^ (Merk Millipore) for 3 weeks and were embedded in paraffin after dehydration. Sections were stained with tartrate-resistant acid phosphatase (TRAP) and TRAP-positive multinucleated ( > 3 nuclei) cells were counted as osteoclasts. To calculate bone loss, the total CEJ–ABC distance of the 12 sites for the control side was subtracted from that of the same 12 sites for ligated side. To explore the cellular source of RANKL in periodontitis-induced bone loss, *Tnfsf11*^*flox/flox*^ mice or *Tnfsf11*^*flox/Δ*^ mice were crossed with Cre lines specific to B cells (*Mb1*-Cre), T cells (*Cd4*-Cre), osteoblastic cells (*Sp7*-Cre), and periodontal ligament cells (*Scx*-Cre). *Sp7*-Cre mice crossed with *Tnfsf11*^*flox/flox*^ mice were treated from the prenatal period with Dox, which was withdrawn at the age of 3 weeks. Periodontitis was induced at the age of 8 weeks and alveolar bone was analyzed 10 days after the ligature placement in all groups. Mice in which the ligatures were lost were excluded from the data.

### Analysis of oral bacterial dissemination

Three-week-old C57BL/6 wild-type female mice were fed with powder food and mice in all of the groups (Control, Ext, PD, PD-Ext) were co-housed for 6 weeks. A 5-0 silk ligature was tied around the maxillary second molar of mice in the PD and PD-Ext groups. The ligature was replaced every week and collected prior to sacrifice at day 42 in the PD group. After ligature placement for 2 weeks, the maxillary second molar was extracted using forceps in Ext and PD-Ext mice under anesthesia. We analyzed the closure of the extraction socket and bacterial colony formation in liver and spleen after 4 weeks of tooth extraction because it was reported that the extraction socket was completely covered by the epithelial tissue in 4 weeks after tooth loss^60^. The healing of oral mucosa and bacterial dissemination after tooth extraction normally finished in *l17a*^*−/−*^*Il17f*^*−/−*^ mice as well as *Il6ra*^*flox/flox*^ *Foxp3*-Cre mice. The liver and spleen were sterilely collected and mechanically homogenized using Tissue homogenizing CKMix (Bertin Technologies) in sterile saline, and cultured on trypticase soy agar with 5% sheep blood (BD Bioscience) under an aerobic condition or CDC anaerobe blood agar (BD Bioscience) under an anaerobic condition for 2 days at 37 °C. The ligature was removed to analyze the bacterial composition under anesthesia just before sacrifice, and bacterial DNA was extracted and analyzed as shown in the following.

### 16S rRNA gene sequencing

Bacterial DNA was extracted from the ligature, fecal samples and tissue cultures with a NucleoSpin^®^ Tissue Kit (Takara). The V4 region of 16 S rRNA genes was PCR amplified (25 cycles, primer pair F515/R806), followed by barcoding (eight cycles) using a Nextera XT Index Kit v2 (Illumina) in triplicate. Barcoded amplicons were sequenced (150-bp paired-end sequencing) on an Illumina MiSeq (Illumina). Sequenced paired-end reads were imported into CLC Genomics Workbench v.9.0 (CLC). Paired-end reads were merged, trimmed, and clustered into operational taxonomic units (OTUs) (97%) by reference-based OTU clustering using Greengenes v13_5. Then the β-diversity was calculated. The primers were as follows: F515, 5′-GTGCCAGCMGCCGCGGTAA-3′ and R806, 5′-GGACTACHVGGGTWTCTAAT-3′. Mice were co-housed for at least 1 week before analysis.

### Evaluation of the total bacterial load

To evaluate the total bacterial load in the oral cavity, the bacterial DNA extracted from ligature was amplified with a Bacteria (*tuf* gene) Quantitative PCR Kit (Takara). Real-time quantitative reverse transcription PCR (RT-PCR) analysis for the *tuf* gene was performed with an initial denaturation step of 95 °C for 30 s, followed by 40 cycles of 95 °C for 5 s, 55 °C for 30 s, and 72 °C for 30 s using SYBR Green (Toyobo) with a LightCycler (Roche). The *tuf* gene copy number was normalized with the ligature length.

### Quantitative RT-PCR analysis

Real-time quantitative RT-PCR analysis was performed with a LightCycler (Roche) using SYBR Green (Toyobo). The level of mRNA expression was normalized with *Gapdh* expression. The following primers were used: *Gapdh*, 5′-TCCACCACCCTGTTGCTGTA-3´and 5′-ACCACAGTCCATGCCATCAC-3′; *Tnfsf11*, 5′-AGCCATTTGCACACCTCAC-3′ and 5′-CGTGGTACCAAGAGGACAGAGT-3′; *Il17a, 5*′-TCCCTCTGTGATCTGGGAAG-3′ and 5′-AGCATCTTCTCGACCCTGAA-3′; *Il17f, 5*′-CAAAACCAGGGCATTTCTGT-3′ and 5′-ATGGTGCTGTCTTCCTGACC-3′; *Il1b, 5*′-CAGGCAGGCAGTATCACTCA-3′ and 5′-AGGTGCTCATGTCCTCATCC-3′; *Il4, 5*′- CCTCACAGCAACGAAGAACA-3′ and 5′- ATCGAAAAGCCCGAAAGAGT-3′; *Ifng, 5*′- GCGTCATTGAATCACACCTG-3′ and 5′- TGAGCTCATTGAATGCTTGG-3′; *Tnfa, 5*′- GCTGAGCTCAAACCCTGGTA-3′ and 5′- CGGACTCCGCAAAGTCTAAG-3′; *Il6, 5*′- CCGGAGAGGAGACTTCACAG-3′ and 5′- CAGAATTGCCATTGCACAAC-3′; *Rorc, 5*′-TGCAAGACTCATCGACAAGG-3′ and 5′- AGGGGATTCAACATCAGTGC-3′; *Defb1, 5*′- AGGTGTTGGCATTCTCACAAG-3′ and 5′- GCTTATCTGGTTTACAGGTTCCC-3′; *Defb4, 5*′- GCAGCCTTTACCCAAATTATC-3′ and 5′- ACAATTGCCAATCTGTCGAA-3′; *Cxcl1*, *5*′- CGCTTCTCTGTGCAGCGCTGCT-3′ and 5′- CAAGCCTCGCGACCATTCTTGA-3′; *Cxcl2, 5*′- TCCAGAGCTTGAGTGTGACG-3′ and 5′- TCCAGGTCAGTTAGCCTTGC-3′. The periodontal tissues were collected from the maxilla as described previously^61^, and homogenized using Tissue homogenizing CKMix (Bertin Technologies) in TRIzol (Life technologies) to extract the total RNA.

### In situ hybridization

Periodontal tissues were fixed with G-Fix (Genostaff), de-calcified with G-Chelate Mild (Genostaff), embedded in paraffin on a CT-Pro20 (Genostaff) using G-Nox (Genostaff) as a less toxic organic solvent for xylene, and sectioned at 5–8 μm. In situ hybridization was performed with the ISH Reagent Kit (Genostaff) according to the manufacturer’s instructions. Tissue sections were de-paraffined with G-Nox, and rehydrated through an ethanol series and phosphate-buffered saline (PBS). The sections were fixed with 10% NBF (10% formalin in PBS) for 30 min at 37 °C and washed in distilled water, placed in 0.2 N HCl for 10 min at 37 °C and washed in PBS, treated with 4 μg ml^−1^ ProteinaseK (Wako Pure Chemical Industries) in PBS for 10 min at 37 °C and washed in PBS, then placed within a coplin jar containing 1xG-Wash (Genostaff), equal to 1xSSC. Hybridization was performed with probes at concentrations of 250 ng ml^−1^ in G-Hybo-L (Genostaff) for 16 h at 60 °C. After hybridization, the sections were washed in 1xG-Wash for 10 min at 60 °C, 50% formamide in 1xG-Wash for 10 min at 60 °C. Then the sections were washed twice in 1xG-Wash for 10 min at 60 °C, twice in 0.1xG-Wash for 10 min at 60 °C and twice in TBST (0.1% Tween 20 in tris-buffered saline (TBS)) at room temperature (RT). After treatment with 1xG-Block (Genostaff) for 15 min at RT, the sections were incubated with anti-DIG AP conjugate (Roche Diagnostics) diluted 1:2000 with x50G-Block (Genostaff) in TBST for 1 h at RT. The sections were washed twice in TBST and then incubated in 100 mM NaCl, 50 mM MgCl_2_, 0.1% Tween 20, 100 mM Tris-HCl, pH 9.5. Coloring reactions were performed with NBT/BCIP solution (Sigma-Aldrich) overnight and then the samples were washed in PBS. The sections were counterstained with Kernechtrot stain solution (Muto Pure Chemicals), and mounted with G-Mount (Genostaff). The probes for *Tnfsf11* and *Il6* were purchased from Genostaff.

### Flow cytometry and antibodies

Antibodies conjugated with biotin, FITC, Alexa Fluor 488, phycoerythrin (PE), PerCP-Cy5.5, allophycocyanin (APC), and pacific blue (PB) were used at a 1:100 dilution unless otherwise mentioned. The following monoclonal antibodies were purchased from eBioscience: anti-human CD2 (RPA-2.10), anti-mouse CD4 (RM4-5), CD25 (PC61), CD39 (24DMS1), CD44 (IM7), CD62L (also called SELL) (MEL-14), CD103 (2E7), OX40 (also called CD134) (OX-86), GITR (also called CD357) (DTA-1, 1:1600), T-cell receptor-β (TCR-β) (H57-597), CCR6 (140706), RANKL (IK22/5), KLRG1 (2F1), FR4 (eBio12A5, 1:400), Foxp3 (FJK-16s), CTLA-4 (UC10-4B9), IFN-γ (XMG1.2), IL-4 (11B11), and IL-17A (eBio17B7). Anti-Helios (22F6) was purchased from BioLegend. Goat anti-mouse/rat Nrp1 (FAB566N, 1:40) was purchased from R&D Systems. The Foxp3 Staining Buffer Set (eBioscience) was used for intracellular Foxp3 staining. For intracellular cytokine staining, cells were stimulated with 50 ng ml^−1^ phorbol myristate acetate (Sigma-Aldrich), 500 ng ml^−1^ ionomycin (Sigma-Aldrich) and GolgiPlug (BD Biosciences) for 5 h. After washing, cells were stained for surface antigens, fixed with 4% paraformaldehyde (Nacalai Tesque) for 10 min at room temperature, and then permeabilized and stained with monoclonal antibodies to cytokine diluted in Perm/Wash buffer (BD Biosciences). Flow cytometric analysis was performed using FACSCanto II with Diva software (BD Biosciences). Periodontal tissues were prepared as described previously^61^. FACS analysis was performed 7 days after placement of the ligature unless otherwise indicated.

### Adoptive transfer

Single-cell suspensions were obtained from peripheral lymph nodes (LNs) and the spleen of periodontitis-induced *Foxp3*^*hCD2*^IL-17-GFP reporter mice 7 days after the ligature placement. Splenic erythrocytes were eliminated with red blood cell lysis buffer (Sigma-Aldrich). To purify the peripheral CD4^+^ T-cell subpopulation, the pooled spleen and LN cells were subjected to a depletion of adherent cells by panning with goat anti-mouse IgG Fc (Cappel, 55472, 1:200) and stained with PE-conjugated mouse anti-human CD2 (RPA-210, eBioscience, 1:100). Cells were then incubated with anti-PE microbeads (Miltenyi Biotech) and separated on LS columns (Miltenyi Biotech). The hCD2^+^ or hCD2^−^ cells were further stained with anti-CD4, anti-CD44, and anti-CD62L monoclonal antibodies, and subjected to FACS sorting using FACSAria III (BD Biosciences). Naive CD44^lo^CD62L^hi^Foxp3^hCD2–^CD4^+^ T cells and CD4^+^ Foxp3^hCD2+^ T cells were collected and then cultured in Iscove’s modified Dulbecco’s medium (Sigma-Aldrich) supplemented with 2 mM l-glutamine, 10% FBS, 50 μM 2-ME, 100 U ml^−1^ penicillin, and 100 μg ml^−1^ streptomycin in the presence of 10 ng ml^−1^ recombinant mouse IL-1β (rmIL-1β) (R&D Systems), 100 ng ml^−1^ rmIL-6 (PeproTech), 50 ng ml^−1^ rmIL-23 (R&D Systems), 5 ng ml^−1^ recombinant human transforming growth factor-β1 (rhTGF-β1) (R&D Systems), 5 μg ml^−1^ anti-IFN-γ (XMG1.2, BD Biosciences), 5 μg ml^−1^ anti-IL-4 (11B11, BD Biosciences) and beads coated with monoclonal antibodies to CD3 and CD28 (Dynal; 25 μl per 1 × 10^6^ cells) for 4 days. Foxp3^hCD2–^IL-17-GFP^+^ cells were then sorted as conventional T_H_17 cells and exFoxp3T_H_17 cells, respectively. The sorted cells were subsequently subjected to adoptive transfer experiments or analysis of their characteristics by FACS and RT-PCR. Adoptive transfer was achieved by a vein injection of 3 × 10^4^ cells into *Tcra*^–/–^ mice just before ligature placement.

### Statistical analyses

Data were analyzed on GraphPad Prism software version 6.0 g. Statistical tests, *n*-values, replicate experiments, and *P*-values are all located in the figures and/or legends. All data are expressed as the mean ± s.e.m. *P*-values were calculated using Student’s *t*-test, analysis of variance (ANOVA) with Dunnett’s or Tukey’s multiple-comparison test, or permutational ANOVA (PERMANOVA) of the weighted UniFrac distance (*P < 0.05; **P < 0.01; ***P < 0.005; NS, not significant, throughout the paper). We estimated the sample size considering the variation and mean of the samples. Neither randomization nor blinding was done in this study. Statistical tests are justified as appropriate for every figure, and the data meet the assumptions of the tests.

### Data availability

Sequence data that support the findings of this study have been deposited in the NCBI Gene Expression Omnibus (GEO) database with the primary accession code GSE109664.

## Electronic supplementary material

Supplementary Information
