# Supplementary material for: Host defense against oral microbiota by bone-damaging T cells
Source: Nat Commun. 2018 Feb 16;9:701. doi: 10.1038/s41467-018-03147-6 (PMC5816021; doi:10.1038/s41467-018-03147-6)
Supplement: Supplementary file 1 — Supplementary Information [file 41467_2018_3147_MOESM1_ESM.pdf]

SUPPLEMENTARY INFORMATION

Tsukasaki *et al.* Host defense against oral microbiota by bone-damaging T cells

Supplementary Figure 1

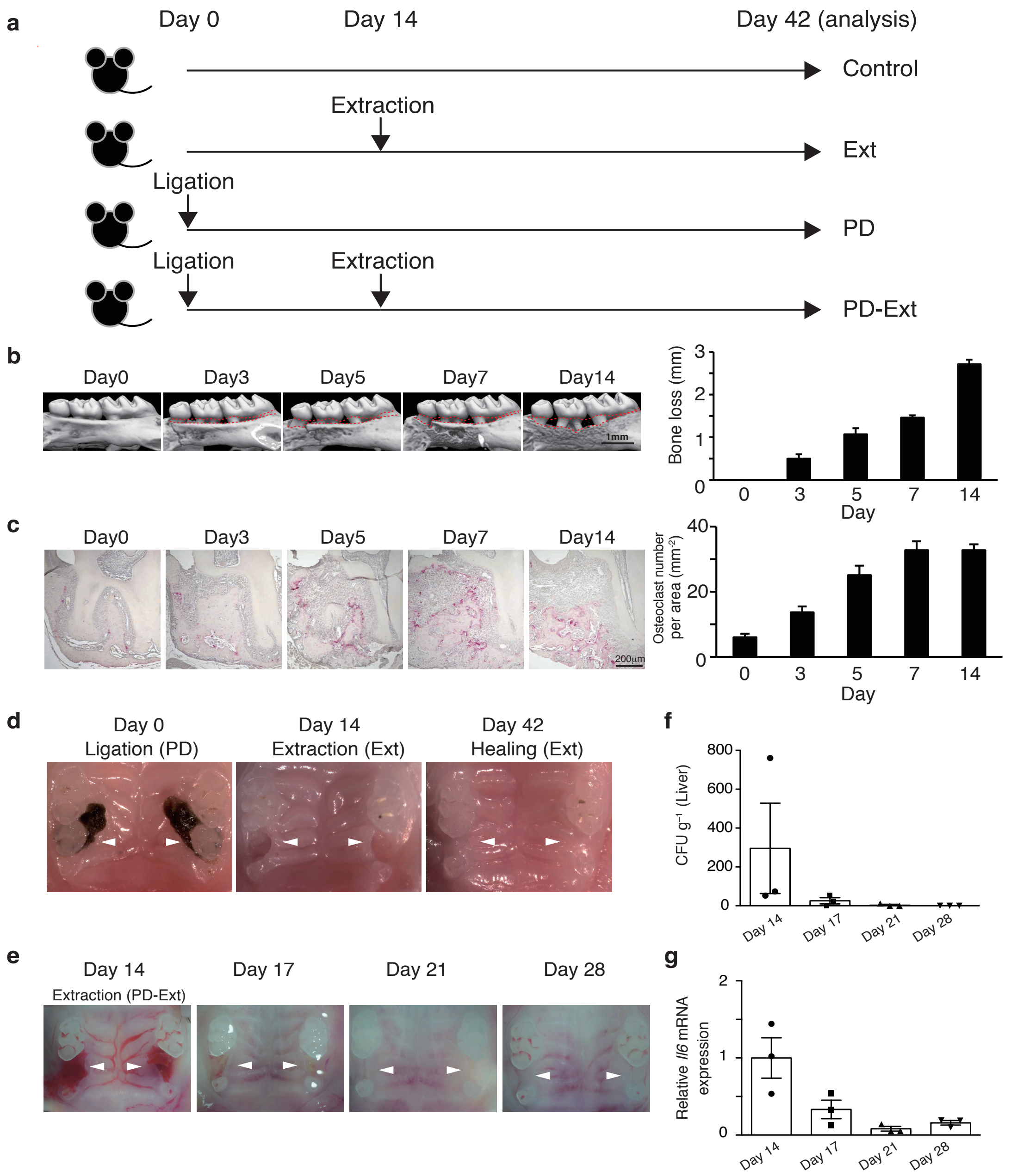

Supplementary Figure 1. Infected-tooth ejection by osteoclastic bone resorption

(a) Schematic of the experimental system for the bacterial colony formation assay on the liver and spleen. **(b)** Micro-CT analysis of periodontitis-induced bone loss in wild-type mice at various time points (0-14 days) after placement of the ligature (n = 2-3). The upper red dotted line indicates the cementoenamel junction and the lower red dotted line indicates the alveolar bone crest. **(c)** Histological analysis of

periodontitis-induced osteoclast development in wild-type mice at various time points (0-14 days) after placement of the ligature (n = 3). **(d)** Ligature placement in PD group (Day 0) and wound healing of the oral mucosa after tooth extraction in Ext group (Day 14 and Day 42). The white arrowheads indicate the second maxillary molar sites. Representative pictures of more than three independent experiments are shown. **(e)** Wound healing of the oral mucosa after tooth extraction in PD-Ext group. The white arrowheads indicate the second maxillary molar sites. Representative pictures of three independent experiments are shown. **(f)** Colony-forming units (CFUs) in aerobic cultures of liver cells from mice in PD-Ext (n = 3) group after tooth extraction. The bacterial dissemination stopped within 1 week after tooth extraction. **(g)** Quantitative RT-PCR analysis of *Il6* transcripts in the periodontal tissues collected from mice in PD-Ext (n = 3) group after tooth extraction. The local inflammation stopped within 1 week after tooth extraction. All data are shown as the mean ± s.e.m.

Supplementary Figure 1 Continued.

Supplementary Figure 2

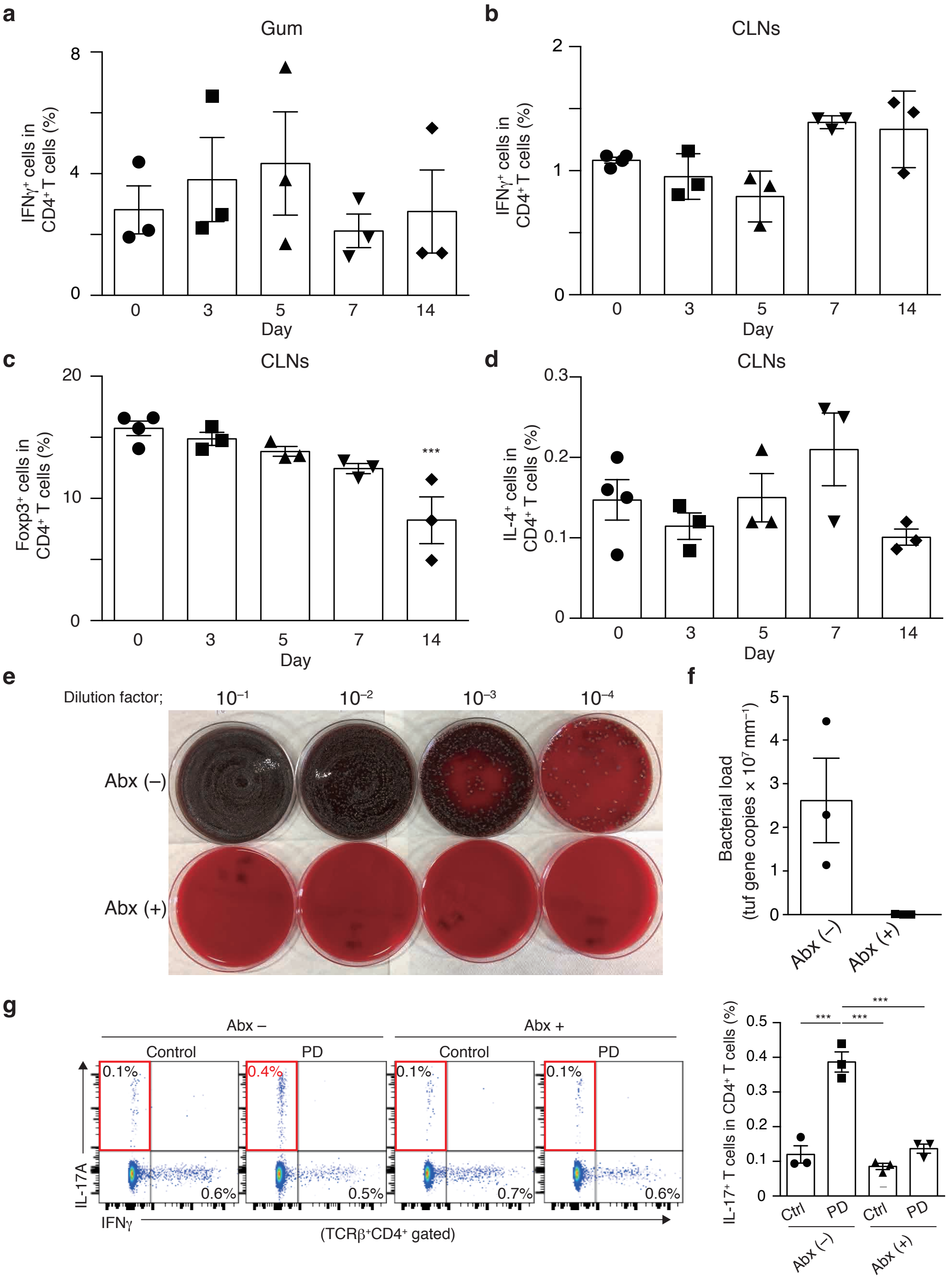

Supplementary Figure 2. Frequency of CD4$^+$ T cell subsets during periodontitis

(a) Frequency of IFNγ$^+$CD4$^+$TCRβ$^+$ cells in the periodontal tissues at various time points (0-14 days) after placement of the ligature (n = 3). **(b)** Frequency of

$IFN\gamma^+CD4^+TCR\beta^+$ cells in the cervical lymph nodes at various time points (0-14 days) after placement of the ligature (n = 3-4). **(c)** Frequency of $Foxp3^+CD4^+TCR\beta^+$ cells in the cervical lymph nodes at various time points (0-14 days) after placement of the ligature (n = 3-4). **(d)** Frequency of $IL\text{-}4^+CD4^+TCR\beta^+$ cells in the cervical lymph nodes at various time points (0-14 days) after placement of the ligature (n = 3-4). **(e and f)** Effects of a broad-spectrum antibiotic cocktail (Abx; ampicillin 1 mg ml^{-1}, streptomycin 5 mg ml^{-1} and colistin 1 mg ml^{-1} in drinking water) on the accumulation of oral bacteria in the ligature. Mice were treated with Abx from one week before placement of the ligature. The ligature was collected one week after the placement and subjected to serial dilutions on aerobic culture (e) or quantitative RT-PCR analysis of the *tuf* gene (f) (n = 3). Oral bacterial accumulation in the ligature was completely inhibited by this antibiotic treatment system. **(g)** Frequency of $IL\text{-}17A^+CD4^+TCR\beta^+$ cells in the cervical lymph nodes were analyzed one week after the ligature placement (n = 3). Ctrl: control; PD: periodontitis. All data are shown as the mean ± s.e.m. Statistical analyses were performed using ANOVA with Dunnett's multiple-comparison test (a-d) and Tukey's multiple-comparison test (g). ***$P < 0.005$.

Supplementary Figure 2 Continued.

Supplementary Figure 3

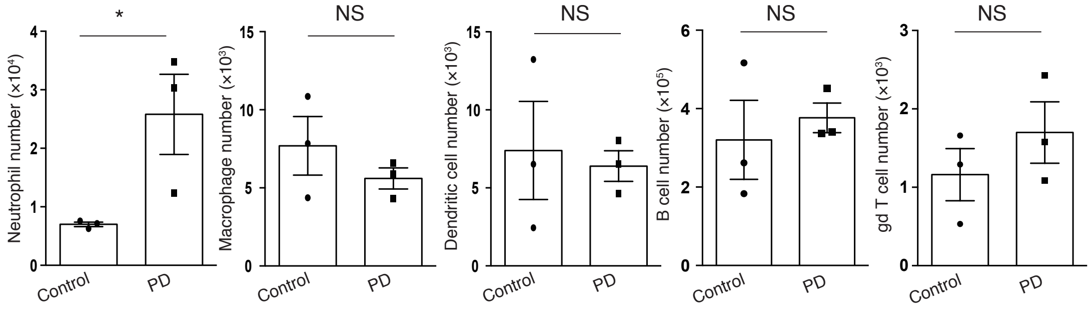

Supplementary Figure 3. Number of the major immune cell subsets in the periodontal tissues

Number of neutrophils ($Gr1^{+}CD11b^{+}$ cells), macrophages ($Gr1^{-}CD11b^{+}F4/80^{+}$ cells), dendritic cells ($CD3^{-}B220^{-}CD11c^{+}MHC2^{+}$ cells), B cells ($CD3^{-}B220^{+}$ cells) and γδT cells ($CD3^{+}B220^{-}TCR\beta^{-}$ cells) in the periodontal tissues in control or periodontitis-induced mice seven days after the ligature placement (n = 3). PD: periodontitis. *$P < 0.05$; NS, not significant.

Supplementary Figure 4

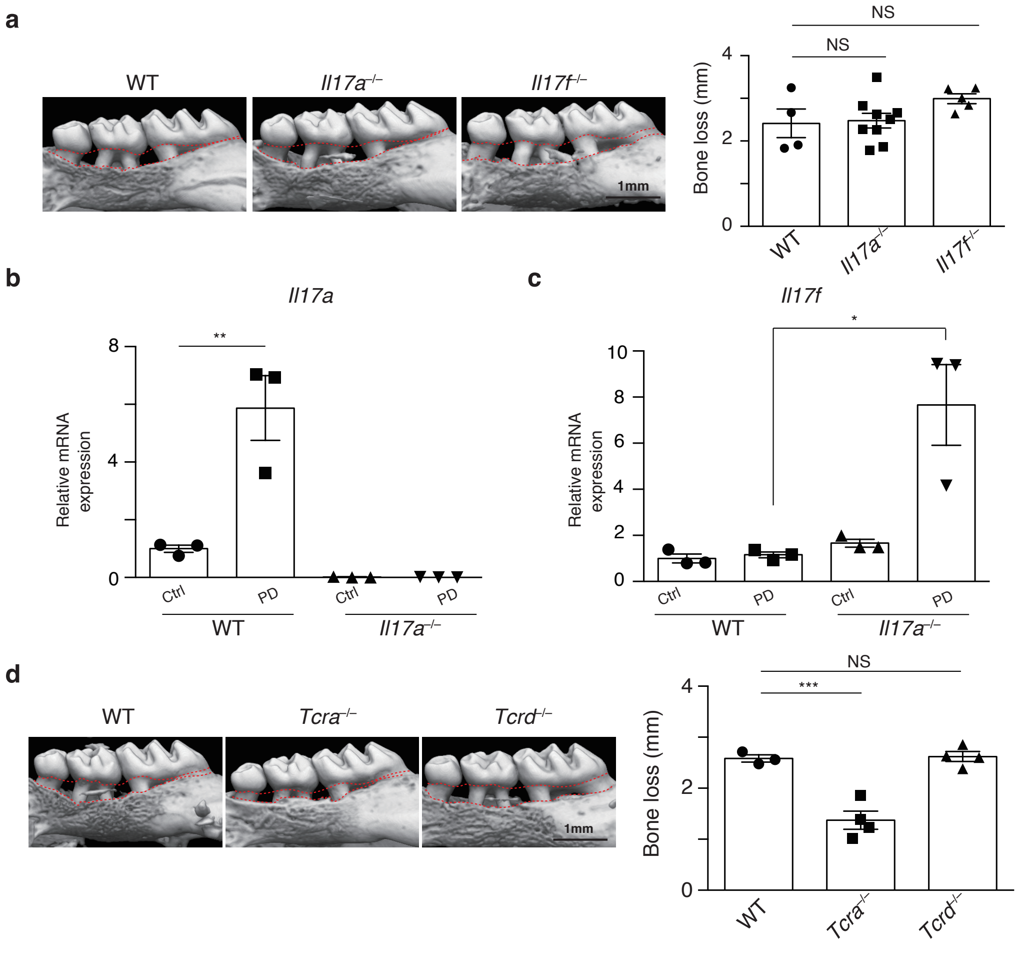

Supplementary Figure 4. The role of IL-17A and IL-17F in periodontitis

(a) Micro-CT analysis of periodontitis-induced bone loss in wild-type mice (n = 4), *Il17a*$^{-/-}$ (n = 9) or *Il17f*$^{-/-}$ mice (n = 5). The upper red dotted line indicates the cementoenamel junction and the lower red dotted line indicates the alveolar bone crest. **(b and c)** Quantitative RT-PCR analysis of *Il17a* and *Il17f* transcripts in the periodontal tissues collected from wild-type mice or *Il17a*$^{-/-}$ mice (n = 3). Ctrl: control; PD: periodontitis. **(d)** Micro-CT analysis of periodontitis-induced bone loss in wild-type mice (n = 3), *Tcra*$^{-/-}$ (n = 4) or *Tcrd*$^{-/-}$ mice (n = 4). The upper red dotted line indicates the cementoenamel junction and the lower red dotted line indicates the alveolar bone crest. The data were pooled from more than two independent experiments (a and d). All data are shown as the mean ± s.e.m. Statistical analyses were performed using ANOVA with Dunnett's multiple-comparison test (a and d) and Student's *t*-test (b and c). $^{*}P < 0.05$; $^{**}P < 0.01$; $^{***}P < 0.005$; NS, not significant.

Supplementary Figure 5

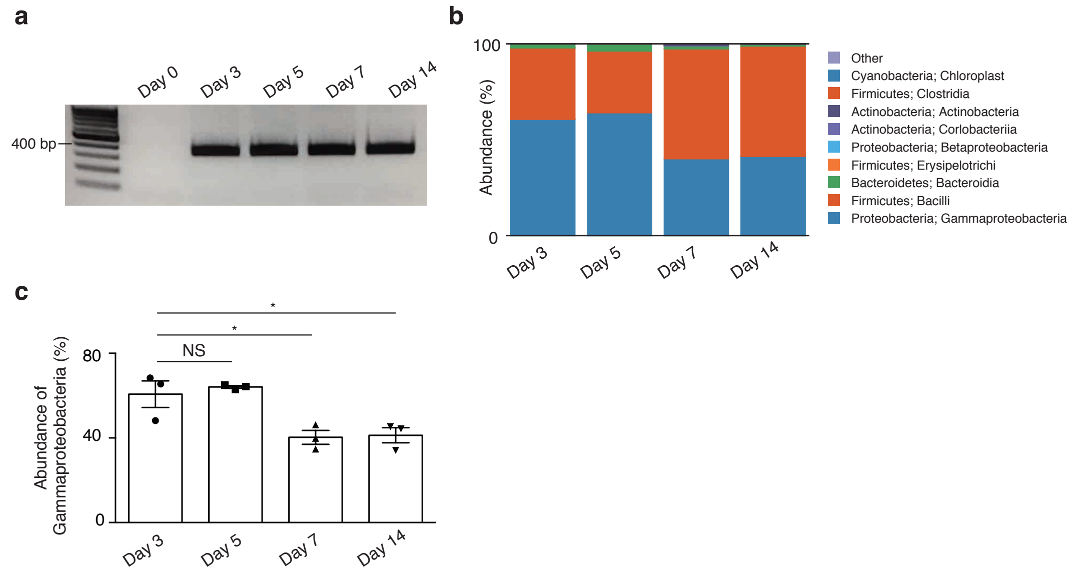

Supplementary Figure 5. The composition of oral bacteria during the course of periodontitis

(a) PCR products of bacterial DNA extracted from the ligatures that were tied around tooth for 30 minutes (Day 0), three days (Day 3), five days (Day 5), seven days (Day 7) or fourteen days (Day 14). The V4 region of 16S rRNA genes was PCR amplified (25 cycles, primer pair F515/R806). PCR analysis failed to detect oral bacteria in Day 0 ligature, indicating that oral bacteria around tooth were too few to be analyzed with 16S sequence analysis in a steady state. Representative data of more than three independent experiments is presented. **(b)** Bacterial composition (major phylum; class) of DNA collected from the ligatures at various time points (3-14 days) after placement of the ligature (n=3) **(c)** The abundance of γ-proteobacteria in the total bacterial DNA collected from the ligatures at various time points (3-14 days) after placement of the ligature (n=3). Statistical analyses were performed using ANOVA with Dunnett's multiple-comparison test (c). *P < 0.05; NS, not significant.

Supplementary Figure 6

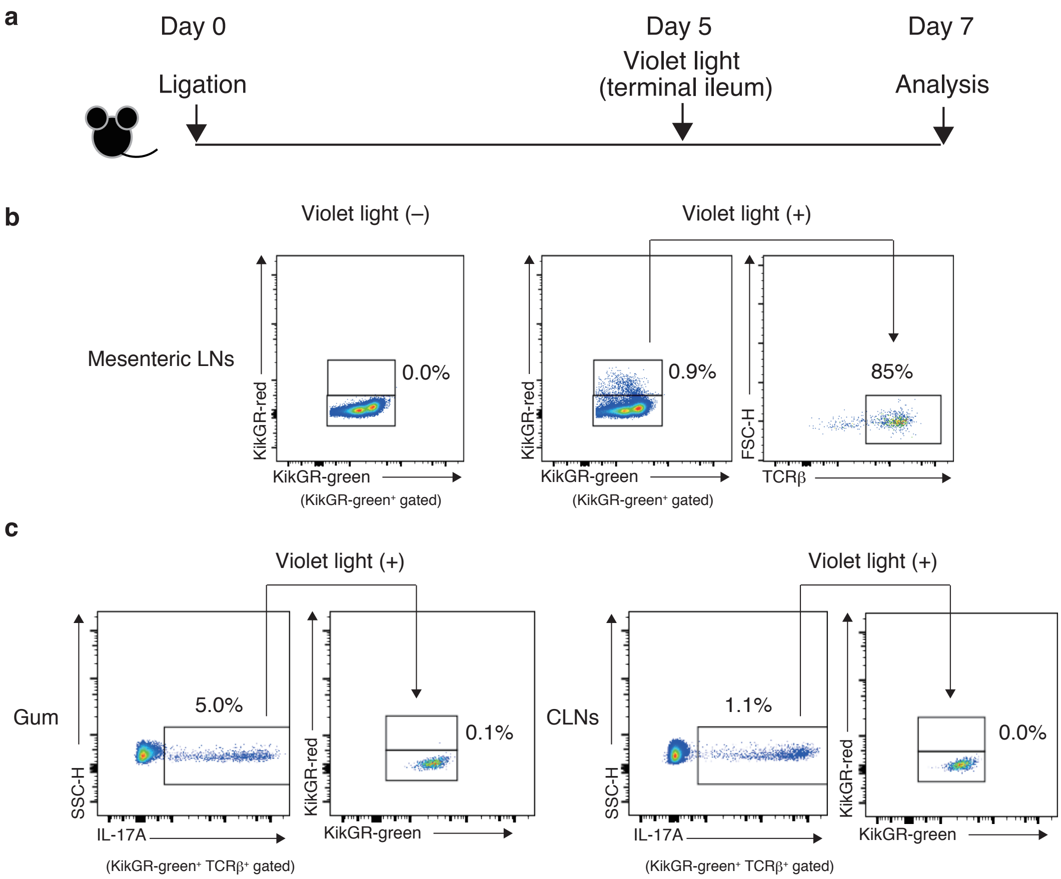

Supplementary Figure 6. T_H17 cells in the periodontal lesion are not originated from the small intestine

(a) Schematic of the experimental system for the immune cell trafficking system using ROSA-CAG-lox-stop-lox-hKikGR × *Vav*-iCre mice. The mucosa of the terminal ileum was exposed to violet light (436 nm) at day 5 under anesthesia, and the mesenteric lymph nodes and the periodontal tissues were collected and analyzed seven days after the ligature placement. **(b)** The frequency of KikGR-red$^+$ cells in the mesenteric lymph nodes. KikGR-red$^+$ cells were not detected in mice which were not exposed to violet light (left panel). KikGR-red$^+$ cells were observed in the mesenteric lymph nodes (LNs) of violet light-exposed mice and most of KikGR-red$^+$ cells were T cells (right panel). **(c)** The frequency of KikGR-red$^+$ cells in the periodontal lesion (Gum) and the cervical lymph nodes (CLNs). T_H17 cells in the periodontal tissues (left panel) or the draining lymph nodes (right panel) were KikGR-red negative. Representative data of more than three independent experiments is shown.

Supplementary Figure 7

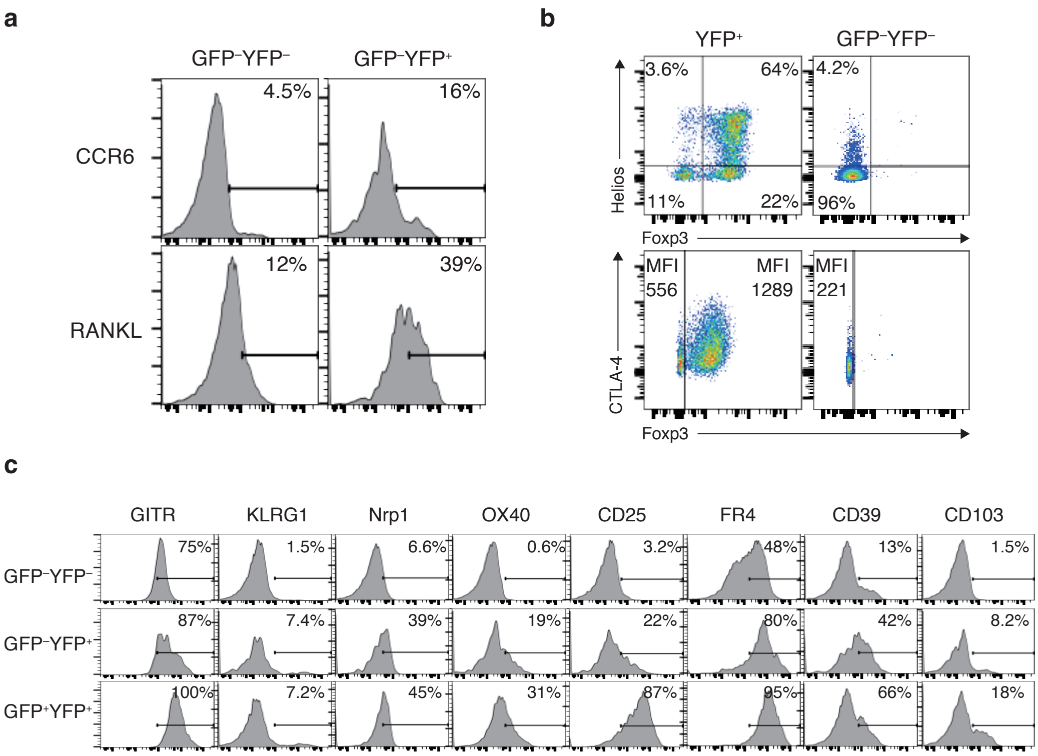

Supplementary Figure 7. Expression of cell surface molecules in exFoxp3 T cells in periodontitis

(a) RANKL and CCR6 expression in GFP^{-}YFP^{-} or GFP^{-}YFP^{+} CD4^{+} T cells of the spleen of *Foxp3*-GFP-Cre × ROSA26-YFP reporter mice at one week after the periodontitis induction. **(b)** YFP^{+} and GFP^{-}YFP^{-} CD4^{+} T cells were sorted from the spleen of *Foxp3*-GFP-Cre × ROSA26-YFP reporter mice one week after the periodontitis induction, and examined for the expression of Foxp3, Helios and CTLA-4. Representative data of two independent experiments are shown. **(c)** Fate mapping analysis of Foxp3-GFP-Cre × ROSA26-YFP reporter mice one week after the periodontitis induction. GFP^{+}YFP^{+}, GFP^{-}YFP^{+} and GFP^{-}YFP^{-} CD4^{+} T cells from the spleen of periodontitis-induced Foxp3-GFP-Cre × ROSA26-YFP reporter mice were examined for the expression of T$_{reg}$ markers. Representative data of two independent experiments is shown (a-c).

Supplementary Figure 8

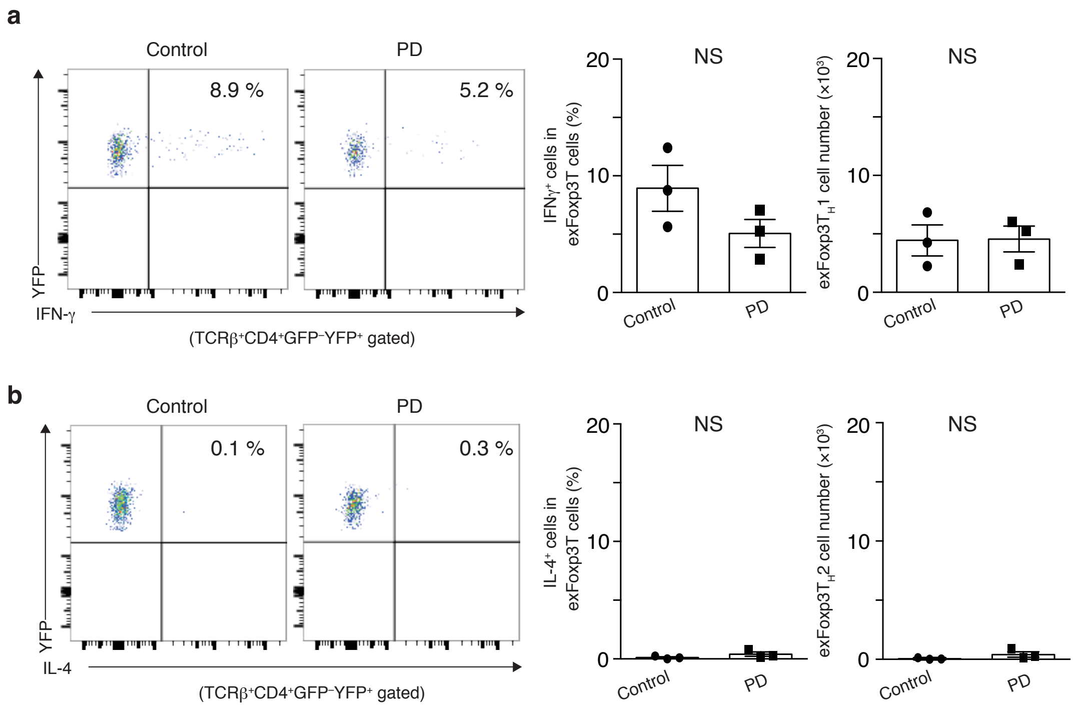

Supplementary Figure 8. Frequency and number of exFoxp3T$_H$1 cells and exFoxp3T$_H$2 cells in periodontitis

(a) Frequency and number of IFNγ$^+$ exFoxp3T cells in the cervical lymph nodes in control or periodontitis-induced Foxp3-GFP-Cre × ROSA26-YFP reporter mice seven days after the ligature placement (n = 3). PD: periodontitis. **(b)** Frequency and number of IL-4$^+$ exFoxp3T cells in the cervical lymph nodes in control or periodontitis-induced Foxp3-GFP-Cre × ROSA26-YFP reporter mice seven days after the ligature placement (n = 3). PD: periodontitis. Statistical analyses were performed using Student's t-test. NS, not significant. The data represents the mean ± s.e.m.

Supplementary Figure 9

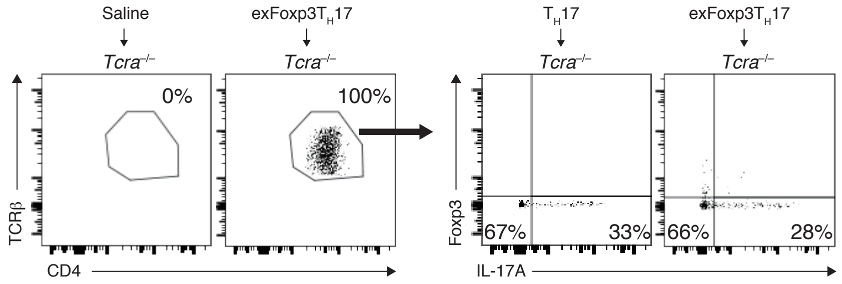

Supplementary Figure 9. Frequency of T_H17 cells and exFoxp3T_H17 cells in *Tcra*$^{-/-}$ mice after the transfer

T_H17 cells or exFoxp3T_H17 cells in the cervical lymph nodes were analyzed for the expression of Foxp3 and IL-17A 10 days after the adoptive transfer. T_H17 cells and exFoxp3T_H17 cells comparably maintained IL-17A expression in the host mice. Representative data of more than three independent experiments is shown.

Supplementary Figure 10

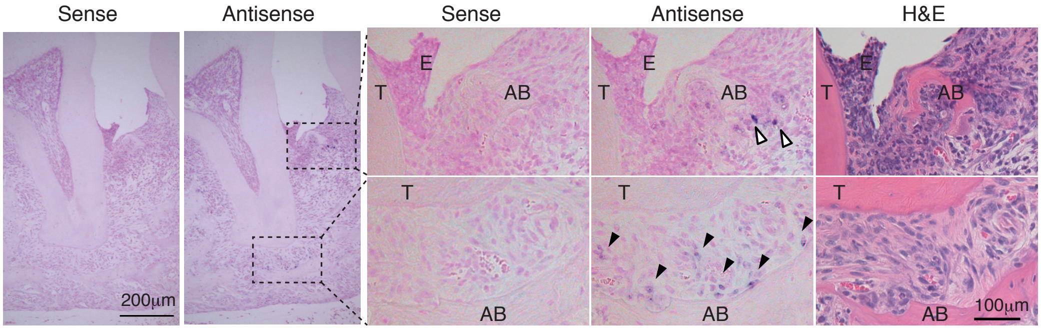

Supplementary Figure 10. RANKL expression in the periodontal lesion

In situ hybridization of *Tnfsf11* mRNA in the periodontal tissues of periodontitis-induced wild-type mice five days after the ligature placement. The white arrowheads indicate RANKL-expressing haematopoietic cells (mononuclear cells present in the inflammatory cell infiltration) adjacent to alveolar bone and the black arrowheads indicate RANKL-expressing mesenchymal cells including osteoblastic cells (cuboidal cells on the alveolar bone surface) and periodontal ligament fibroblasts (spindle-shape cells connecting tooth to the alveolar bone). H&E: haematoxylin and eosin stain, E: epithelium; T: tooth; AB: alveolar bone. Representative data of more than three independent experiments is shown.

Supplementary Figure 11

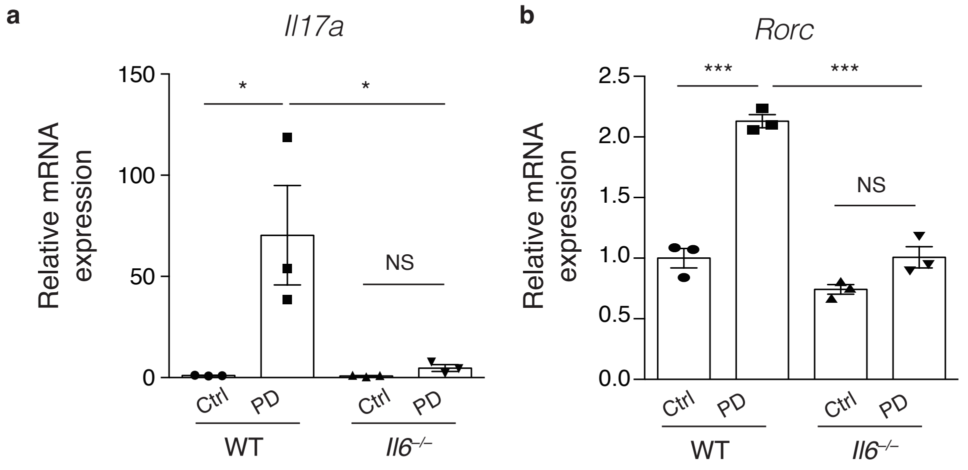

Supplementary Figure 11. Expression of *Il17* and *Rorc* in wild-type or *Il6*$^{-/-}$ mice during periodontitis

(a) Quantitative RT-PCR analysis of *Il17a* transcripts in the periodontal tissues collected from wild-type mice or *Il6*$^{-/-}$ mice (n = 3). The periodontal tissues were collected 10 days after the ligature placement. Ctrl: control; PD: periodontitis. **(b)** Quantitative RT-PCR analysis of *Rorc* transcripts in the $CD4^+$ T cells collected from cervical lymph nodes of wild-type mice or *Il6*$^{-/-}$ mice (n = 3). The $CD4^+$ T cells were collected 10 days after the ligature placement. Ctrl: control; PD: periodontitis. All data are shown as the mean ± s.e.m. Statistical analyses were performed using Student's *t*-test. *$P < 0.05$; ***$P < 0.005$; NS, not significant.

Supplementary Figure 12

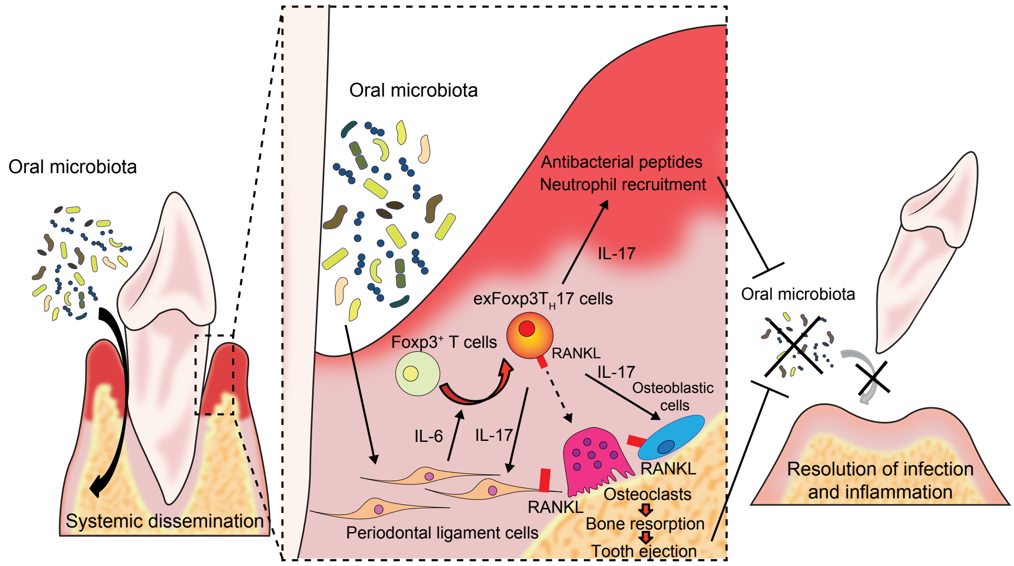

Supplementary Figure 12. Schematic model of the host defense system against oral bacteria

Oral microbiota systemically disseminate via the inflamed gingiva during periodontitis. Oral microbial invasion leads to local inflammation and IL-6 production by periodontal ligament cells. IL-6 facilitates the conversion of Foxp3$^+$ T cells into exFoxp3T$_H$17 cells. exFoxp3T$_H$17 cells produce IL-17, which contributes to the eradication of oral microbiota by inducing the expression of antibacterial peptides and neutrophil chemo-attractants in the gingival epithelial cells. exFoxp3T$_H$17 cells also promote osteoclastic bone resorption mainly by inducing RANKL expression on osteoblastic cells and periodontal ligament cells via IL-17 production. Tooth loss caused by bone resorption may have been beneficial in terminating the systemic dissemination of oral microbiota. Thus, bone-damaging T cells play a crucial role in the anti-bacterial response.
